# 2-Bromo-3-nitro­benzaldehyde

**DOI:** 10.1107/S160053680904104X

**Published:** 2009-10-17

**Authors:** Vijay P. Singh, Harkesh B. Singh, Ray J. Butcher

**Affiliations:** a Department of Chemistry, Indian Institute of Technology Bombay, Powai, Mumbai 400 076, India; b Department of Chemistry, Howard University, 525 College Street NW, Washington, DC 20059, USA

## Abstract

The title compound, C_7_H_4_BrNO_3_, was isolated as a by-product while attempting to prepare a diselenide. There is a close intra­molecular Br⋯O contact [2.984 (2) Å]. The mol­ecules form loosely associated dimers held together by weak inter­molecular Br⋯O inter­actions with the nitro O atoms [Br⋯O = 3.179 (3) Å]. As a result of these inter­actions, there is also a close Br⋯Br inter­molecular contact [3.8714 (6) Å]. In addition, there are weak inter­molecular C—H⋯O inter­actions. The combination of these inter­actions produces sheets which propagate in the (210) and (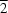
10) directions perpendicular to *c*.

## Related literature

For the preparation and reactivity of the title compound, see: Rahman & Scrowston (1984 ▶); Sienkowska *et al.* (2000 ▶); Wirth & Fragale (1997 ▶). For bond-length data, see: Allen (2002 ▶). For intramolecular chalcogen interactions, see: Singh *et al.* (2009 ▶). For intermolecular Br⋯O interactions, see: Jones & Lozano (2004 ▶); Kruszynski (2007 ▶); Pedireddi *et al.* (1992 ▶);  Xie *et al.* (2009 ▶).
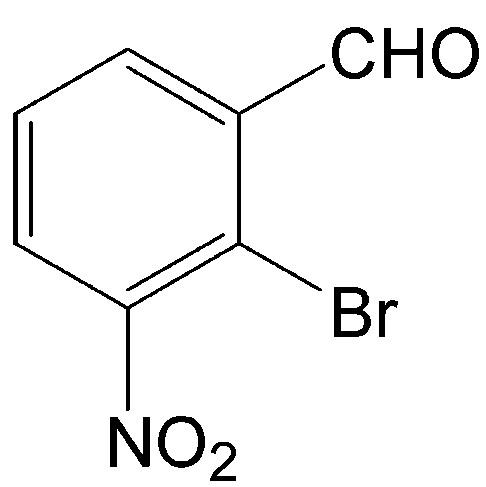


## Experimental

### 

#### Crystal data

C_7_H_4_BrNO_3_
*M* *_r_* = 230.02
Monoclinic, 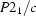

*a* = 8.1578 (8) Å
*b* = 6.3079 (5) Å
*c* = 15.0537 (11) Å
β = 91.603 (8)°
*V* = 774.34 (11) Å^3^
*Z* = 4
Mo *K*α radiation
μ = 5.27 mm^−1^
*T* = 296 K
0.27 × 0.18 × 0.09 mm

#### Data collection

Oxford Diffraction Gemini R diffractometer
Absorption correction: multi-scan (*CrysAlisPro*; Oxford Diffraction, 2009 ▶) *T*_min_ = 0.330, *T*_max_ = 0.649
5208 measured reflections
2120 independent reflections
1308 reflections with *I* > 2σ(*I*)
*R*_int_ = 0.031

#### Refinement

*R*[*F* ^2^ > 2σ(*F* ^2^)] = 0.031
*wR*(*F* ^2^) = 0.079
*S* = 0.97
2120 reflections
109 parameters
H-atom parameters constrained
Δρ_max_ = 0.77 e Å^−3^
Δρ_min_ = −0.45 e Å^−3^

### 

Data collection: *CrysAlisPro* (Oxford Diffraction, 2009 ▶); cell refinement: *CrysAlisPro*; data reduction: *CrysAlisPro*; program(s) used to solve structure: *SHELXS97* (Sheldrick, 2008 ▶); program(s) used to refine structure: *SHELXL97* (Sheldrick, 2008 ▶); molecular graphics: *SHELXTL* (Sheldrick, 2008 ▶); software used to prepare material for publication: *SHELXTL*.

## Supplementary Material

Crystal structure: contains datablocks I, global. DOI: 10.1107/S160053680904104X/om2283sup1.cif

Structure factors: contains datablocks I. DOI: 10.1107/S160053680904104X/om2283Isup2.hkl

Additional supplementary materials:  crystallographic information; 3D view; checkCIF report

## Figures and Tables

**Table 1:** Hydrogen-bond geometry (Å, °)

*D*—H⋯*A*	*D*—H	H⋯*A*	*D*⋯*A*	*D*—H⋯*A*
C6—H6⋯O1^i^	0.93	2.55	3.354 (4)	145
C7—H7⋯O3^ii^	0.93	2.62	3.534 (4)	168

Symmetry codes: (i) 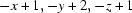; (ii) 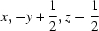.

**Fig. 1.**
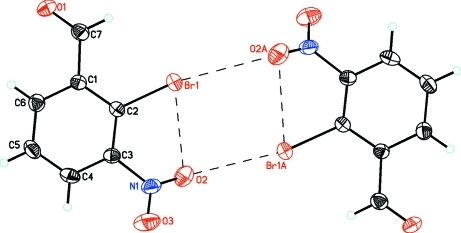
The molecular structure of C7H4NO3Br the showing the Br···O intra- and intermolecular interactions (as dashed lines) forming loosely associated dimers. The atom numbering scheme and 50% probability displacement ellipsoids.

**Fig. 2.**
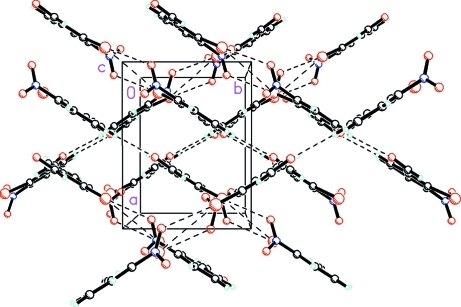
The molecular packing for C7H4NO3Br viewed down the c axis showing the sheets of associated molecules in the (2 1 0) and (-2 1 0) directions. The secondary interactions are shown by dashed lines.

**Fig. 3.**
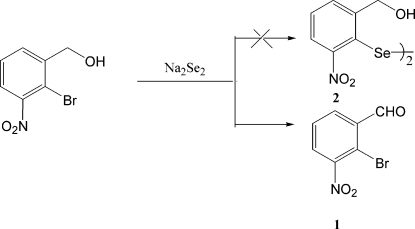
The formation of the title compound.

## supplementary crystallographic information

### Comment

The stucture of the title compound, (I), is shown below. Dimensions are available in the archived CIF.

The title compound 1, C_7_H_4_NO_3_Br, was isolated as a by-product while attempting to prepare diselenide 2 by reacting 2-bromo-3-nitrobenzylalcohol with disodium diselenide (Wirth & Fragale, 1997) as shown in scheme 1. Presumably, the formation of 1 takes place during column chromatography on silica gel where the alcohol function is oxidized to the aldehyde function. The preparation (but not the structure) of the title compound by different routes has been previously reported (Rahman & Scrowston, 1984; Sienkowska *et al.*, 2000). In 1, with two withdrawing *ortho* groups present, the 2-position is highly susceptible to nucleophilic substitution by Na_2_Se_2_, Na_2_Te_2_, Na_2_Se to afford a series of novel chalcogen compounds (Singh *et al.* 2009). In this paper we report the structure of the precursor.

The bond lengths and angles in the title compound are within the normal ranges for related compounds (Allen *et al.*, 2002). When chalcogens (Se, Te) are present in the 2-position in place of bromine there is an intramolecular chalcogen (Se/Te···oxygen(aldehyde/nitro)) interaction (Singh *et al.* 2009). It was of interest to see whether the bromo analog will interact intramolecularly with the nitro/aldehyde donor groups. There is a close intramolecular Br···O contact of 2.984 (2) Å. The molecules form loosely associated dimers held together by weak intermolecular Br···O interactions with the nitro O atoms (Br···O 3.179 (3) Å, see Figure 1). Similar interactions have been previously reported (Jones & Lozano, 2004; Kruszynski, 2007; Pedireddi *et al.*, 1992; Xie *et al.*, 2009). As a result of these interactions there is also a close Br···Br intermolecular contact (3.8714 (6) Å) as has been commonly observed [42 examples found in a search of the Cambridge Structural Database (Allen, 2002)]. In addition there are weak intermolecular C—H···O interactions. Of the intermolecular interactions, only that between O3 and the aldehyde H is out of plane. As a result of this out-of-plane interaction the nitro group is twisted by 43.6 (4)° from the plane of the aromatic ring. The combination of these interactions produces sheets which propagate in the (2 1 0) and (-2 1 0) directions perpendicular to c as shown in Figure 2.

### Experimental

The title compound 1, C_7_H_4_NO_3_Br, was isolated as a by-product while attempting to prepare diselenide 2 by reacting 2-bromo-3-nitrobenzylalcohol with disodium diselenide (Wirth & Fragale, 1997). Presumably, the formation of 1 takes place during column chromatography on silica gel where the alcohol function is oxidized to the aldehyde function. It has been prepared previously by a different routes (Rahman & Scrowston, 1984; Sienkowska *et al.* 2000).

Crystal suitable for X-ray diffraction were obtained from CH_2_Cl_2_/ethyl acetate.

### Crystal data

**Table:**

C_7_H_4_BrNO_3_	*F*(000) = 448
*M**_r_* = 230.02	*D*_x_ = 1.973 Mg m^−^^3^
Monoclinic, *P*2_1_/*c*	Mo *K*α radiation, λ = 0.71073 Å
*a* = 8.1578 (8) Å	Cell parameters from 2069 reflections
*b* = 6.3079 (5) Å	θ = 4.7–30.5°
*c* = 15.0537 (11) Å	µ = 5.27 mm^−^^1^
β = 91.603 (8)°	*T* = 296 K
*V* = 774.34 (11) Å^3^	Rectangular plate, orange
*Z* = 4	0.27 × 0.18 × 0.09 mm

### Data collection

**Table:**

Oxford Diffraction Gemini R diffractometer	2120 independent reflections
Radiation source: Enhance (Mo) X-ray Source	1308 reflections with *I* > 2σ(*I*)
graphite	*R*_int_ = 0.031
Detector resolution: 10.5081 pixels mm^-1^	θ_max_ = 30.6°, θ_min_ = 4.9°
ω scans	*h* = −11→10
Absorption correction: multi-scan (*CrysAlis PRO*; Oxford Diffraction, 2009)	*k* = −8→7
*T*_min_ = 0.330, *T*_max_ = 0.649	*l* = −21→11
5208 measured reflections	

### Refinement

**Table:**

Refinement on *F*^2^	Primary atom site location: structure-invariant direct methods
Least-squares matrix: full	Secondary atom site location: difference Fourier map
*R*[*F*^2^ > 2σ(*F*^2^)] = 0.031	Hydrogen site location: inferred from neighbouring sites
*wR*(*F*^2^) = 0.079	H-atom parameters constrained
*S* = 0.97	*w* = 1/[σ^2^(*F*_o_^2^) + (0.0388*P*)^2^] where *P* = (*F*_o_^2^ + 2*F*_c_^2^)/3
2120 reflections	(Δ/σ)_max_ < 0.001
109 parameters	Δρ_max_ = 0.77 e Å^−^^3^
0 restraints	Δρ_min_ = −0.45 e Å^−^^3^

### Special details

**Table:**

Geometry. All e.s.d.'s (except the e.s.d. in the dihedral angle between two l.s. planes) are estimated using the full covariance matrix. The cell e.s.d.'s are taken into account individually in the estimation of e.s.d.'s in distances, angles and torsion angles; correlations between e.s.d.'s in cell parameters are only used when they are defined by crystal symmetry. An approximate (isotropic) treatment of cell e.s.d.'s is used for estimating e.s.d.'s involving l.s. planes.
Refinement. Refinement of *F*^2^ against ALL reflections. The weighted *R*-factor *wR* and goodness of fit *S* are based on *F*^2^, conventional *R*-factors *R* are based on *F*, with *F* set to zero for negative *F*^2^. The threshold expression of *F*^2^ > σ(*F*^2^) is used only for calculating *R*-factors(gt) *etc*. and is not relevant to the choice of reflections for refinement. *R*-factors based on *F*^2^ are statistically about twice as large as those based on *F*, and *R*- factors based on ALL data will be even larger.

### Fractional atomic coordinates and isotropic or equivalent isotropic displacement parameters (Å^2^)

**Table:**

	*x*	*y*	*z*	*U*_iso_*/*U*_eq_	
Br1	0.14664 (4)	0.22483 (5)	0.465488 (18)	0.04777 (13)	
O1	0.4218 (3)	0.7906 (3)	0.39513 (14)	0.0541 (5)	
O2	−0.0180 (3)	0.1829 (4)	0.63917 (17)	0.0744 (7)	
O3	0.1725 (4)	0.1401 (5)	0.73881 (17)	0.0868 (8)	
N1	0.1141 (4)	0.2271 (4)	0.67396 (17)	0.0505 (7)	
C1	0.3125 (3)	0.6054 (4)	0.51753 (16)	0.0348 (6)	
C2	0.2254 (3)	0.4311 (4)	0.54696 (16)	0.0328 (5)	
C3	0.2073 (3)	0.4060 (4)	0.63756 (17)	0.0370 (6)	
C4	0.2767 (3)	0.5454 (5)	0.69788 (18)	0.0470 (7)	
H4	0.2653	0.5227	0.7584	0.056*	
C5	0.3621 (4)	0.7169 (5)	0.6692 (2)	0.0508 (8)	
H5	0.4088	0.8119	0.7098	0.061*	
C6	0.3785 (4)	0.7478 (4)	0.5787 (2)	0.0434 (7)	
H6	0.4348	0.8660	0.5587	0.052*	
C7	0.3397 (4)	0.6456 (5)	0.42181 (18)	0.0443 (7)	
H7	0.2910	0.5545	0.3803	0.053*	

### Atomic displacement parameters (Å^2^)

**Table:**

	*U*^11^	*U*^22^	*U*^33^	*U*^12^	*U*^13^	*U*^23^
Br1	0.0561 (2)	0.04590 (19)	0.04115 (18)	−0.00979 (14)	−0.00130 (13)	−0.00726 (13)
O1	0.0632 (13)	0.0526 (13)	0.0468 (12)	−0.0092 (11)	0.0069 (10)	0.0147 (10)
O2	0.0641 (16)	0.0862 (18)	0.0735 (17)	−0.0285 (14)	0.0124 (14)	0.0052 (14)
O3	0.119 (2)	0.0817 (19)	0.0591 (15)	−0.0035 (17)	−0.0007 (15)	0.0315 (15)
N1	0.0631 (18)	0.0518 (16)	0.0375 (13)	−0.0010 (13)	0.0145 (13)	0.0043 (12)
C1	0.0361 (14)	0.0354 (14)	0.0329 (13)	0.0038 (11)	0.0010 (11)	−0.0006 (11)
C2	0.0306 (13)	0.0351 (13)	0.0325 (13)	0.0046 (11)	−0.0014 (10)	−0.0021 (11)
C3	0.0363 (14)	0.0407 (14)	0.0341 (14)	0.0034 (12)	0.0039 (11)	0.0007 (11)
C4	0.0520 (18)	0.060 (2)	0.0291 (13)	0.0078 (15)	0.0055 (13)	−0.0049 (13)
C5	0.0549 (19)	0.0558 (18)	0.0413 (16)	−0.0046 (15)	−0.0044 (14)	−0.0159 (14)
C6	0.0449 (17)	0.0392 (17)	0.0460 (16)	−0.0018 (12)	0.0017 (13)	−0.0033 (13)
C7	0.0486 (17)	0.0447 (16)	0.0394 (15)	0.0044 (14)	−0.0053 (13)	0.0003 (13)

### Geometric parameters (Å, °)

**Table:**

Br1—C2	1.889 (2)	C1—C7	1.486 (4)
Br1—O2	2.984 (2)	C2—C3	1.385 (3)
Br1—Br1^i^	3.8714 (6)	C3—C4	1.375 (4)
O1—C7	1.209 (3)	C4—C5	1.363 (4)
O2—N1	1.217 (4)	C4—H4	0.9300
O2—Br1^i^	3.179 (3)	C5—C6	1.387 (4)
O3—N1	1.206 (4)	C5—H5	0.9300
N1—C3	1.475 (4)	C6—H6	0.9300
C1—C6	1.384 (4)	C7—H7	0.9300
C1—C2	1.388 (3)		
			
C2—Br1—O2	69.23 (9)	C4—C3—C2	121.6 (2)
C2—Br1—Br1^i^	122.15 (8)	C4—C3—N1	116.8 (2)
O2—Br1—Br1^i^	53.36 (5)	C2—C3—N1	121.6 (2)
N1—O2—Br1	86.66 (16)	C5—C4—C3	120.2 (3)
N1—O2—Br1^i^	132.4 (2)	C5—C4—H4	119.9
Br1—O2—Br1^i^	77.75 (6)	C3—C4—H4	119.9
O3—N1—O2	124.7 (3)	C4—C5—C6	119.1 (3)
O3—N1—C3	116.9 (3)	C4—C5—H5	120.4
O2—N1—C3	118.4 (3)	C6—C5—H5	120.4
C6—C1—C2	119.6 (2)	C1—C6—C5	121.1 (3)
C6—C1—C7	117.9 (2)	C1—C6—H6	119.5
C2—C1—C7	122.4 (2)	C5—C6—H6	119.5
C3—C2—C1	118.3 (2)	O1—C7—C1	123.4 (3)
C3—C2—Br1	121.12 (19)	O1—C7—H7	118.3
C1—C2—Br1	120.42 (18)	C1—C7—H7	118.3
			
C2—Br1—O2—N1	−37.66 (19)	Br1—C2—C3—C4	174.2 (2)
Br1^i^—Br1—O2—N1	134.7 (2)	C1—C2—C3—N1	178.9 (2)
C2—Br1—O2—Br1^i^	−172.41 (10)	Br1—C2—C3—N1	−5.2 (3)
Br1—O2—N1—O3	−137.5 (3)	O3—N1—C3—C4	−41.7 (4)
Br1^i^—O2—N1—O3	−67.4 (4)	O2—N1—C3—C4	135.4 (3)
Br1—O2—N1—C3	45.6 (2)	O3—N1—C3—C2	137.7 (3)
Br1^i^—O2—N1—C3	115.6 (3)	O2—N1—C3—C2	−45.1 (4)
C6—C1—C2—C3	0.2 (4)	C2—C3—C4—C5	1.8 (4)
C7—C1—C2—C3	179.5 (2)	N1—C3—C4—C5	−178.8 (3)
C6—C1—C2—Br1	−175.8 (2)	C3—C4—C5—C6	−0.3 (4)
C7—C1—C2—Br1	3.6 (3)	C2—C1—C6—C5	1.3 (4)
O2—Br1—C2—C3	19.93 (19)	C7—C1—C6—C5	−178.1 (3)
Br1^i^—Br1—C2—C3	12.7 (2)	C4—C5—C6—C1	−1.3 (5)
O2—Br1—C2—C1	−164.3 (2)	C6—C1—C7—O1	3.1 (4)
Br1^i^—Br1—C2—C1	−171.45 (16)	C2—C1—C7—O1	−176.3 (3)
C1—C2—C3—C4	−1.7 (4)		

Symmetry codes: (i) −*x*, −*y*, −*z*+1.

### Hydrogen-bond geometry (Å, °)

**Table:**

*D*—H···*A*	*D*—H	H···*A*	*D*···*A*	*D*—H···*A*
C6—H6···O1^ii^	0.93	2.55	3.354 (4)	145
C7—H7···O3^iii^	0.93	2.62	3.534 (4)	168

Symmetry codes: (ii) −*x*+1, −*y*+2, −*z*+1; (iii) *x*, −*y*+1/2, *z*−1/2.
